# Defining research priorities for youth public mental health: reflections on a coproduction approach to transdisciplinary working

**DOI:** 10.1186/s12961-022-00871-w

**Published:** 2022-06-20

**Authors:** Andrea Taylor, Christina McMellon, Tara French, Alice MacLachlan, Rhiannon Evans, Ruth Lewis, Mark McCann, Laurence Moore, Simon Murphy, Sharon Simpson, Jo Inchley

**Affiliations:** 1 School of Design, Glasgow School of Art, Glasgow, UK; 2 MRC/CSO Social and Public Health Science Unit, University of Glasgow, Glasgow, UK; 3 Innovation School, Glasgow School of Art, Forres, UK; 4 Centre for Development, Evaluation, Complexity and Implementation in Public Health Improvement (DECIPHer), Cardiff University, Cardiff, UK

**Keywords:** Coproduction, Mental health, Public health, Research priorities, Transdisciplinary, Young people

## Abstract

**Background:**

With most mental health problems established during childhood/adolescence, young people must be a key focus of public mental health approaches. Despite the range of factors known to influence mental health, evidence for effective interventions is lacking for this age group. This study aimed to define priorities for future public health intervention-focused research to support youth mental health by engaging with transdisciplinary stakeholder groups.

**Methods:**

Our coproduction approach involved priority-setting workshops with young people, researchers, practitioners and policy-makers. Each workshop focused on three thematic areas: social connections and relationships; schools and other education settings; and key groups at greater risk of mental ill-health, specifically LGBTQ+ and care-experienced young people. Workshop outputs were synthesized to define research priorities.

**Results:**

This paper presents the research priorities that were defined through the priority-setting workshops, and our reflections on the coproduction approach to guide future similar activities undertaken by others. Ten priorities for youth public mental health research were defined, covering the following areas: building supportive relationships; whole system approaches; social media; support at times of transition; improving links between different services; development and training for those who support young people; staff mental health; engaging with families; awareness of and access to services; and out-of-school and community settings.

**Conclusions:**

These research priorities can inform future intervention development to support youth public mental health. Our transdisciplinary approach means the identified research priorities are likely to be relevant to young people’s experiences and needs, and to fit with the needs of those working in practice and policy to support young people.

## Background

The mental health of young people (defined as those aged 10–24 years [1]) is a major global public health challenge [2] and a national priority in the United Kingdom [3], with 75% of mental health problems established by age 24 [4]. With greater understanding of the risk and protective factors for mental health that span individual, community, organizational and societal levels, mental health is increasingly being recognized as a public health issue [5]. Public mental health strategies focus on the promotion of good mental health and prevention of mental ill-health at the population level, while also targeting those who are at greatest risk and who may not benefit from universal approaches [6, 7]. Public mental health strategies also highlight a life course approach. However, evidence for effective interventions to promote good mental health and prevent mental ill-health among young people remains limited. Young people’s lives are complex and diverse, and their mental health may be impacted by a range of different factors, including the added impact of recent disruption to their lives caused by the COVID-19 pandemic. Evidence is needed to support interventions aiming to improve young people’s mental health, but with a wide range of different domains that could be targeted through public health approaches, it is important to prioritize those areas that are most relevant to young people and where intervention is most welcome and feasible.

### A transdisciplinary approach to identifying priority areas for research

In order to ensure that interventions to support youth public mental health are relevant to young people, and to those who support young people, it is important that their voices are included when making decisions on the areas to prioritize within research. Collaboration between representatives of different disciplines across academic, public, private and voluntary sectors as well as public involvement to share knowledge on an issue and work together to develop new research and identify solutions is known as a transdisciplinary approach [8]. However, bringing together different groups of stakeholders to achieve a common aim is not without challenges, and requires a collaborative approach to facilitate the sharing of knowledge and experiences and support different groups to work together effectively [8]. Coproduction and design-led approaches have been highlighted as effective methods to facilitate transdisciplinary working [9].

Coproduction was originally developed as an approach to service development where adult service users were recognized as active participants in shaping and delivering services rather than as passive recipients of services [10]. In recent years, coproduction has become a popular approach in development work with young people and, most recently, in health research [11]. Within mental health research specifically, coproduction is a relatively new and innovative approach, with evidence of positive involvement and outcomes including improved mental well-being for people involved in the process [10, 12]. The New Economics Foundation has developed six principles of coproduction that are widely used as a framework to understand coproduction [10]:

1. Taking an assets-based approach: transforming the perception of people, so that they are seen not as passive recipients of services and burdens on the system, but as equal partners in designing and delivering services.
2. Building on people’s existing capabilities: altering the delivery model of public services from a deficit approach to one that provides opportunities to recognize and grow people’s capabilities and actively support them to put these to use at an individual and community level.
3. Reciprocity and mutuality: offering people a range of incentives to work in reciprocal relationships with professionals and with each other, where there are mutual responsibilities and expectations.
4. Peer support networks: engaging peer and personal networks alongside professionals as the best way of transferring knowledge.
5. Blurring distinctions: removing the distinction between professionals and recipients, and between producers and consumers of services, by reconfiguring the way services are developed and delivered.
6. Facilitating rather than delivering: enabling public service agencies to become catalysts and facilitators rather than being the main providers themselves.

### Defining research priorities for youth public mental health

While previous priority-setting work for mental health research exists (e.g. [13, 14]), only a few studies have engaged with stakeholders, including young people, to wholly focus on defining research priorities for young people’s mental health. These include a United Kingdom-based study by the McPin Foundation taken forward by the National Institute for Health and Care Research (NIHR), a more global study by the New Zealand-based Children and Young People Satellite, and an England-based study by the Emerging Minds mental health network [15].

The McPin study [16] used an established process called the James Lind Alliance (JLA) Priority Setting Partnership (PSP) [17]. JLA PSPs bring patients, carers and clinicians together in priority-setting partnerships to set the Top 10 priority areas for research in particular areas of health and care [17]. The JLA PSP process includes the following: creating a steering group to take responsibility for the PSP, with equal representation from patients, carers and clinicians; a survey asking patients, carers and clinicians what questions they have for research and a literature search to find evidence gaps; summarizing the survey responses into a long list of summary questions and removing any questions that have already been answered by research; a second survey asking patients, carers and clinicians to vote on the most important questions; and a workshop to discuss the highest ranked 25–30 questions and agree on the Top 10 list of priorities [18]. The results of the McPin study highlighted a range of topics around interventions and services for young people’s mental health such as early screening of mental health problems and waiting times for services. The process aligns with coproduction through its emphasis on equal relationships between patients, carers and clinicians. However, the process does not include developing the priorities into specific research questions. Subsequently, the NIHR identified and prioritized research questions within the 10 areas identified by the McPin study, using a health research prioritization method pioneered by the Child Health and Nutrition Research Initiative (CHNRI) [19]. The typical CHNRI process includes creating a team to manage the process and set criteria for a research idea/question to be considered a research priority; asking a large number of researchers to contribute research ideas/questions and to then score them against each criterion; and inviting external stakeholders (e.g. patients and carers) to set different thresholds and weights for each of the criteria, giving some criteria greater importance than others. The final output of the CHNRI process is a list that ranks up to 200 research ideas/questions by their scores against the criteria [19]. However, contrary to coproduction, much of the process is controlled by funders and researchers. Indeed, a lack of good stakeholder involvement was reported across the first 50 applications of the CHNRI method, possibly due to difficulty implementing the method as envisaged [20]. The results of the NIHR exercise are currently confidential and being written up for publication.

The Children and Young People Satellite, hereafter referred to as the Satellite, is an offshoot of the Cochrane Common Mental Disorders Review Group, which is itself a part of Cochrane, whose mission is to provide high-quality information to support health decisions [21]. As part of a large programme of work to establish and prioritize research questions for children and young people’s mental health, the Satellite distributed an online survey to stakeholders, with particular effort directed towards including children and young people with mental health problems [22]. The survey was in two sections. The first section involved ranking a list of common mental disorders and difficulties that cause functional impairments. The second section included open-text questions to identify factors that increase children and young people’s vulnerability to poor mental health and potential ways to address them. Results showed respondents believe anxiety, depression and suicide are the most important mental health problems to be addressed, and highlighted the need for service reforms, and strengthening parenting and educational responses as important ways to address mental disorders. As with the CHNRI method described above, the approach taken by the Satellite is very different to coproduction, with stakeholders taking part in the study as research participants only.

Emerging Minds identified four research challenges through a priority-setting process that began with stakeholder engagement workshops. The YoungMinds charity led three workshops with children and young people, and parents/carers [23]. Then, the Centre for Mental Health charity led four workshops with practitioners and policy-makers [24]. The following four research challenges were identified: The Big Question—implementing effective interventions at scale; Embracing Complexity—supporting children and young people who have intersecting needs and face complex situations; Voices, Power and Attitudes—amplifying the voices and power of children and young people, and changing attitudes about mental health; and Supporting the Supporters—supporting children and young people themselves and those around them. The Emerging Minds workshop approach aligns with coproduction through its emphasis on taking an assets-based approach and involving stakeholders at the beginning of the process. Reports of the workshops are available [23, 24], although a detailed description of the full priority-setting process has not yet been published.

Importantly, the focus of the McPin, NIHR and Satellite studies tend towards treatment and support for those with diagnosed mental health problems, rather than public mental health approaches and related interventions that focus on promotion and prevention at a population level. The Emerging Minds research also has a focus on reducing the prevalence of mental health problems, although with an emphasis on promotion and prevention as well as treatment. This paper describes the coproduction approach undertaken by the Transdisciplinary Research for the Improvement of Youth Mental Public Health (TRIUMPH) Network to define research priorities for youth public mental health, with a focus on identifying key areas for intervention development. The TRIUMPH Network is one of eight UK Research and Innovation (UKRI)-funded United Kingdom-based mental health research networks that has a particular focus on research to improve young people’s mental health [25]. Central to the TRIUMPH Network’s priority-setting process was the active involvement of a range of stakeholders including young people, academics, practitioners and policy-makers from across different public and mental health-related disciplines.

From a public mental health perspective, whilst recognizing the broad range of influences on young people’s mental health, the TRIUMPH Network identified three thematic areas as a focus for further research based on the expert knowledge of members of the study team and existing evidence in this field: Key Groups of young people at greater risk of mental ill-health—initially focusing on care-experienced and lesbian, gay, bisexual, transgender and queer/questioning (LGBTQ+) young people [26, 27]; Social Connections and Relationships [28–33]; and Schools and Other Education Settings [34]. These thematic areas reflect key social and contextual factors that affect young people’s mental health, rather than individual-psychological factors that are more common in traditional mental health research.

This paper presents the priorities and specific questions for research on young people’s mental health that were defined through the priority-setting process within each of the three thematic areas. Using the New Economics Foundation principles [10], we also reflect on the coproduction approach that was taken to facilitate transdisciplinary working and ensure a wide range of stakeholders, including young people, were actively involved in the priority-setting process.

## Methods

While coproduction provided the overarching approach for involving stakeholders, a design-led approach, supportive of the key principles of coproduction and building on the expertise of the TRIUMPH Network team, was used to structure engagement with stakeholders and facilitate conversations. The approach for identifying research priorities for youth public mental health had three main stages: (1) a priority-setting workshop with young people from the TRIUMPH Network Youth Advisory Group (YAG; described below); (2) priority-setting workshops and online consultation with TRIUMPH Network stakeholders from across the United Kingdom; (3) collation of workshop/consultation outputs and identification of final research priorities. All TRIUMPH Network activities have been approved by the University of Glasgow College of Social Sciences Research Ethics Committee (reference: 400180214). Participants were informed that the information gathered from the workshops would be used to identify research priorities for the TRIUMPH Network. By attending the workshops, participants consented to their data being used for this purpose.

### Stage 1: Priority-setting with the TRIUMPH Network YAG

Within the TRIUMPH Network, a key mechanism for involving young people as research partners is via YAG. The YAG’s role is to support the development of the Network, including being involved in strategic decision-making. The YAG is made up of 16 young people aged 16–24 years, recruited through four partner youth organizations, one in each nation of the United Kingdom, who were chosen to reflect the whole population remit of the Network and also the two identified key groups of young people (those with care experience and LGBTQ+) [35]. YAG members are reimbursed for their time and contribution to the Network in line with NIHR guidelines [36].

#### Workshop

**Fig. 1:**
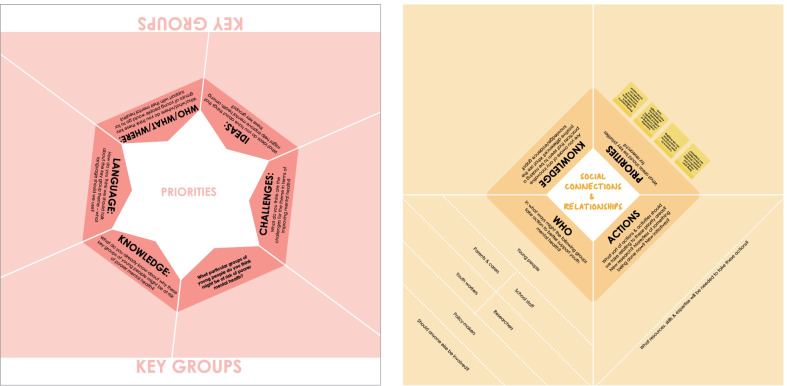
Examples of conversation templates used with the YAG (left) and wider stakeholder groups (right)

The priority-setting process began in June 2019 with a workshop involving all 16 members of the YAG. Three workshop tables were set up, one per thematic area, with a large table-top conversation template that was designed to structure discussion and guide facilitators to engage the YAG in sharing their insights. The template was divided into the following six key questions: Knowledge—about the thematic area; Experience—of the thematic area; Challenges—for the thematic area in terms of improving mental health; Language—that the TRIUMPH Network should use in relation to the thematic area; Who/what—young people would go to for support with their mental health; and Ideas—for how to improve mental health within the thematic area (see Fig. 1 left). Working in three groups, and on a rotational basis, each group spent 30 min at each table discussing the assigned thematic area. The discussions were facilitated by academic researchers from the TRIUMPH Network with expert knowledge of each thematic area (hereafter called “theme leads”) and captured on Post-it Notes and attached to the relevant section of the template. After discussing all three thematic areas, the YAG members were each given three stickers to collaboratively identify their top/preferred priorities for each thematic area that they would most like to see progressed. The group was advised that they could use more than one dot for a single item (Post-it Note) if they felt strongly about it.

Following the workshop, two researchers from the TRIUMPH Network team analysed the young people’s discussions through emergent clustering and synthesis of the Post-it Note contributions. For each thematic area, the researchers clustered the Post-it Notes individually and reviewed the resulting topics together. The theme leads provided further contextualized detail on the synthesized list of topics based on the discussions they had facilitated around these topics. Members of the YAG subsequently reviewed and agreed on the final list of priorities from the workshop to confirm that these accurately reflected and captured their discussions and ideas. The priorities identified by the YAG were carried forward into stage 2 of the priority-setting process.

### Stage 2: United Kingdom-wide stakeholder priority-setting

#### Workshops

Four priority-setting workshops were conducted across the United Kingdom—in Belfast, Cardiff, Glasgow and London—between November 2019 and January 2020. Academics, practitioners and policy-makers from different sectors including health and social care, education and third-sector organizations with relevant expertise in young people’s mental health were identified and invited to attend. Local youth organizations and schools were also contacted to invite/support young people to attend the workshops. A total of 188 people participated in the workshops (Belfast = 41; Cardiff = 55; Glasgow = 44; London = 28). Of these, 20.2% (*n* = 38) were young people aged 13–24 years, 35.6% (*n* = 67) were academics, 29.3% (*n* = 55) were practitioners and 14.9% (*n* = 28) were policy-makers. Disciplines included those with expertise in public health, psychiatry, psychology, neuroscience, education, healthcare, social work, youth work and art and design. Prior to the workshops, participants were sent a brief about each theme, which included a theme summary, key insights from the YAG priority-setting workshop and questions for exploration in the workshops.

**Fig. 2:**
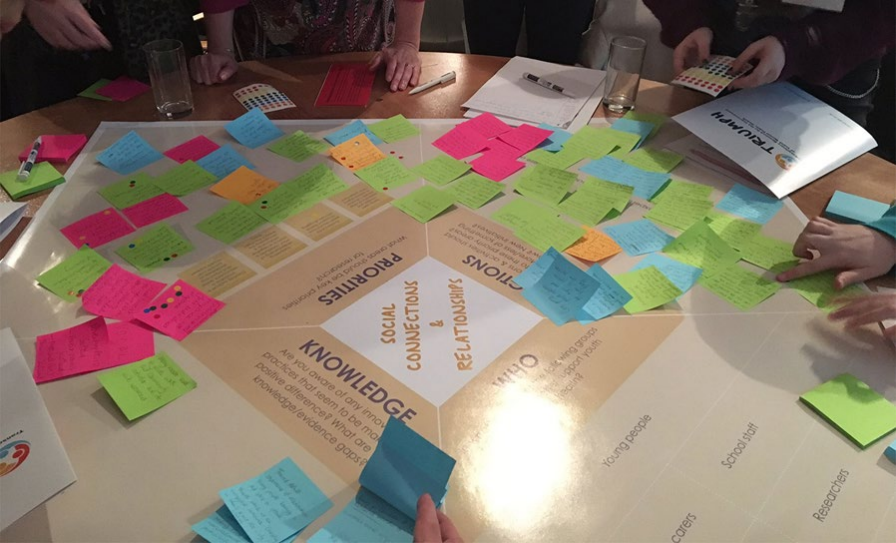
Example of workshop attendees capturing discussions using the conversation template

The main workshop session followed a similar format to the YAG workshop. Tables were organized by thematic area, and each participant was involved in discussions for two thematic areas (of their choice and relevant to their experience), which lasted approximately 1 hour each. Each table discussion was led by a facilitator (typically a theme lead) and supported by a dedicated Post-it Note-taker, and an updated version of the YAG workshop conversation template was used to guide the discussion (see Fig. 1, right and Fig. 2). The template was divided into four sections with the following key questions: Knowledge—of innovative practices that are making a positive difference and knowledge/evidence gaps; Priorities—identification of areas that should be key priorities for research; Actions—that can be taken relating to these priority areas; and Who—ways in which particular groups of people can take those actions. Importantly, the Priorities section of the template included the priorities previously identified at the YAG workshop for that thematic area.

#### Online consultation

In parallel with the workshops, a short online survey was conducted. The survey included questions on views on the key factors influencing young people’s mental health and what needs to change to improve young people’s mental health. Respondents were asked to identify key research questions or topics they think need to be addressed within each of the TRIUMPH Network thematic areas in order to make these changes. The survey was publicly accessible and was advertised through the TRIUMPH Network mailing list and Twitter. Of the 46 people who completed the survey, three were young people, 17 were academics, 23 were practitioners and three were policy-makers, although respondents were able to select more than one of these categories in their response, and some did not complete this section of the survey.

### Stage 3: Analysis of workshop/consultation outputs and identification of research priorities

The Post-it Note contributions from each of the stage 2 workshops were transcribed and organized based on thematic area and United Kingdom nation, and the contributions from the online consultation were incorporated. The collective data were then analysed by four researchers from the TRIUMPH Network team. Each researcher was assigned a thematic area (Key Groups was separated into two: care-experienced and LGBTQ+ young people). Within each thematic area, the researchers clustered the data collected from the Priorities area of the conversation template (including the YAG priorities), along with the data from the online consultation, into draft topics (research priorities). The researchers then came together to review across all thematic areas to identify recurring and discrete priorities, that is, priorities that were identified across all thematic areas or specific to just one or two areas. Then, a subset of the Post-it Notes contributions from each of the other three discussion areas of the conversation template (Knowledge, Actions and Who) were cross-checked against the identified priorities to ensure there were no additional topics that had not already emerged and to validate the identified priorities.

The results of the clustering analysis were reviewed by the YAG who agreed with the content but suggested alternative wording of some priorities. The draft priorities, together with the full results of the thematic clustering, were then reviewed by the theme leads. The theme leads ensured the priorities were within the scope of public mental health research and organized the broad list of specific priorities into a reduced set of overarching priority areas, with a subset of research questions within each priority area that collectively allowed scope for the full/original list of priorities to be addressed. A key focus of the theme leads was in translating identified priorities for outcomes/action within policy and practice into questions that could be addressed by research. The wording and structure of the research priorities and questions were then revised for consistency across the three thematic areas. A final review took place between the researchers involved in the original clustering analysis to ensure nothing had been lost without good reason.

#### Reflection process

Reflections on the coproduction approach to engagement and involvement of multiple stakeholders in identifying research priorities involved a combination of organic and structured reflection among the first four authors. Organic reflection involved regular reflection during and after workshops which informed the methods and approach in subsequent workshops. Structured reflection was prompted through the development of this paper and based on the New Economics Foundation’s coproduction principles [10] to reflect on both the process and experiential learning of the authors. This process was also informed by evaluation received from stakeholders who participated in the workshops; however, this was limited and varied across groups.

## Results

This section presents the research priorities that were defined through the priority-setting workshops with young people, researchers, practitioners and policy-makers, and our reflections on the coproduction approach that was taken to facilitate transdisciplinary working to guide future similar activities undertaken by others.

### YAG priorities for mental health

Within each of the TRIUMPH Network thematic areas, young people identified priorities where they felt improvements should be made to support their mental health (Table 1).

**Table 1:** YAG priorities to support mental health

Key Groups
•Training and education for parents/carers, teachers and young people about issues faced by key groups (e.g. LGBTQ+-inclusive sex education in schools) •Ensuring an equal amount of awareness was given to different key groups •Removing jargon so that conversations around key groups are more accessible and easier to understand •Ensuring mental health is seen separately from identity for young people within key groups and not judging people based on how different aspects of their identities intersect •Providing better access to mental health support services (e.g. victim support services; improved Child and Adolescent Mental Health Services [CAMHS] waiting times)
Social Connections and Relationships
•Guidance for peers around how to support friends who are struggling •Supporting young people to understand what constitutes a positive or negative relationship and recognizing the impact this might have on their mental health •Ensuring support is available to young people and improving their awareness so people can access support •Being open-minded and respectful of other people’s situation to reduce stigma and “judgement”
Schools andOther Education Settings
•Training and education on mental health for both teachers and young people (e.g. through personal and social education programmes) •Better support for young people with mental health problems (e.g. through peer support and regular check-ins) •Better communication to young people from the education setting on its policies and procedures for mental health •Effective anti-bullying policies •Less comparison between young people •Provide extra support for high achievers

### Final research priorities for youth public mental health

A final set of 10 priorities for research into youth mental public health were defined through the analysis of the United Kingdom-wide stakeholder priority-setting workshops (incorporating the priorities from the YAG priority-setting workshop) and responses to the online consultation. The priorities are as follows:

- Building relationships that support good mental health and well-being;
- Whole system approaches to support young people’s mental health;
- Social media and mental health;
- Supporting young people at times of transition;
- Improving links between different services and settings;
- Development and training for those who support young people’s mental health;
- Staff mental health and well-being;
- Engaging with families;
- Young people’s awareness, access and experience of services;
- Out-of-school and community settings that support mental health and well-being.

Each of these priorities was discussed across all of the TRIUMPH Network thematic areas to some extent; however, within each thematic area different evidence gaps and research questions were identified with some priorities more prominent in some thematic areas than others. The specific research questions highlighted within each thematic area are presented in Tables 2, 3 and 4.

**Table 2:** Final research priorities for Social Connections and Relationships

Building positive relationships that support good mental health and well-being •How can we support young people to develop positive peer relationships, relationships with parents/carers, and relationships with other adults (e.g. teachers, youth workers)? •What is the role of relationships in young people’s resilience to respond to change and adversity?
Whole system approaches to support young people’s mental health •How can we change culture to reduce stigma and support mental health and well-being? •How can we reduce the impact of inequalities (e.g. deprivation, social inequalities, geographical area) on mental health and well-being?
Social media and mental health •How can we harness social media in a positive way to support mental health and well-being?
Out of school and community settings to support mental health and well-being •What is the role of youth clubs, social prescribing (i.e. referrals to local non-clinical community services) and outdoor activities in supporting youth mental health? •What skills/support do parents and carers need in order to support young people and how can this be provided?

**Table 3:** Final research priorities for Schools and Other Education Settings

Building positive relationships that support good mental health and well-being •What role do schools play in developing relationship education?
Whole school system approaches to support young people’s mental health and well-being •How can we change culture around well-being and attainment (e.g. policies, curriculum)? •How could the school curriculum be modified to support young people’s mental health? •How can changes to the physical or structural education environment affect mental health and well-being (e.g. class size, timetable structure, built environment, physical activity, time outdoors)? •How can we develop mental health literacy among young people to increase awareness and reduce stigma around mental health and well-being? •How do we measure change in whole school/education setting environments?
Social media and mental health •How can education settings support the digital literacy of students and staff?
Supporting young people at times of transition •How can we support young people to develop the skills and resilience to adapt to changes in their lives (e.g. moving from primary to secondary to tertiary education, starting work, or moving in and out of different education settings)? •What additional support should education settings provide to support young people through transitions?
Improving links to mental health services and support •What processes and policies are needed to allow interagency working linked to education settings that provides consistent and appropriate support for young people? •How should young people in education settings be involved in decision-making around mental health services and support?
Development and training for those who support young people’s mental health and well-being •What are the development needs of education staff (e.g. teachers, support staff, mental health specialists in education settings) in order to provide appropriate support to young people? •What training is effective for staff in education settings? •How can training on mental health and well-being be successfully implemented in education settings?
Staff mental health and well-being •What do staff in education settings need to support their own mental health and well-being? •How can we effectively implement strategies or interventions that support staff well-being?
Engaging with families •How can education settings effectively engage with families, particularly parents/carers, to support them in understanding young people mental health issues? •How can families work with education to provide a consistent support system for young people both in and out of the education setting?

**Table 4:** Final research priorities for Key Groups

LGBTQ+ young people
Building positive relationships that support good mental health and well-being •How can we support LGBTQ+ young people to develop supportive peer networks that are protective of mental health? •How can we support LGBTQ+ young people to develop positive relationships within families, wider communities, and with social care, healthcare and education professionals? •What is the role of relationships in LGBTQ+ young people’s resilience to respond to change and adversity?
Whole system approaches to support LGBTQ+ young people’s mental health and well-being •How can we change culture to reduce stigma relating to LGBTQ+ identities and lives, and support mental health? •How can we achieve positive and sustainable change in schools and communities that supports mental health among young people of all genders and sexualities? •How can we increase positive representations of LGBTQ+ identities, relationships and lives in popular and mainstream culture to improve mental health among LGBTQ+ young people?
Supporting LGBTQ+ young people through change •What are the potentially challenging periods of change in LGBTQ+ young people’s lives and how do these impact mental health (e.g. moving from primary to secondary to tertiary education, leaving home, starting employment, healthcare/service changes)? •How can we best support LGBTQ+ young people during these periods of change?
Development and training for those who support LGBTQ+ young people •What are the training needs of adults (e.g. in education, youth work, healthcare, social care) supporting the mental health of LGBTQ+ young people? •What training outcomes are important to LGBTQ+ young people for adults working to support them? •How should LGBTQ+ young people be involved in decision-making around training and support?
Care-experienced young people
Building relationships and networks that support good mental health and well-being •How can we support care experienced young people to develop supportive peer networks? •How can we support young people and their carers to develop positive relationships that are protective of mental health? •How can we support care experienced young people to develop positive relationships with social care, healthcare and education professionals?
Supporting care-experienced young people at times of transition •What are the key transitions for care-experienced young people and how do these impact mental health (e.g. in and out of care, leaving care, between services, educational transitions)? •What are the challenges and opportunities in achieving positive transitions, and how can care-experienced young people be supported in managing these transitions?
Models of interagency working to support mental health and well-being •What are the needs of social care, healthcare and education professionals who support care-experienced young people with their mental health? •What are the opportunities and challenges of different models to support interagency working? •What are the impacts of different models to support interagency working on young people’s mental health? •How can we implement successful models of interagency working across different settings?
Development and training for those who support care-experienced young people •What are the training needs of adults (e.g. social care, healthcare, education, carers) supporting the mental health of care-experienced young people? •What training outcomes are important to adults and care-experienced young people? •How can mental health training be successfully implemented among adults who support care-experienced young people?
Awareness, access and experience of services •What are the service needs of care-experienced young people to support their mental health? •What are care-experienced young people’s experiences of current mental health services and support and how could this be improved? •How can we increase awareness and access to mental health services and support among care-experienced young people? What are the potential impacts on inequalities?

### Reflections on the priority-setting approach

Here, we return to the New Economics Foundation’s coproduction principles [10] to reflect upon our process and learning.

#### Taking an assets-based approach

Through coproduction of the TRIUMPH Network’s research priorities, we ensured that young people, practitioners and policy-makers were involved early on as partners in developing the research agenda. This is important because young people and other stakeholders are often brought into the research process after the research topic and questions have been defined by academics. Through involving stakeholders early on in the process, we value and validate the knowledge, skills and experience that they bring to the work. Stakeholder engagement during the research process may also facilitate the translation of research into policy and practice [37].

#### Building on people’s existing capabilities

Although all of the stakeholder groups are viewed as equal partners, the TRIUMPH Network has a clear process for building young people’s capacity to participate in research processes, in the form of the YAG, whereas processes to involve practitioners and policy-makers are less structured. This is reflected in the involvement of different stakeholder groups in the priority-setting process where YAG members had multiple points where they could influence the priorities, whereas practitioner and policy-maker involvement was largely limited to the priority-setting workshops and online consultation. The coproduction approach and stakeholders’ views clearly influenced the final research priorities. For example, at the first workshop, the YAG members provided feedback on the language used to discuss the different aspects of their lives and suggested changes in the names of the thematic areas, which were subsequently updated as suggested, and many of the final research priorities reflect the practice-based focus of the priority-setting workshop discussions among practitioners and policy-makers.

#### Reciprocity and mutuality

This principle speaks to the transdisciplinary nature of the TRIUMPH Network, where research collaboratives are built not only across academic disciplines but also across different stakeholder groups. YAG members provided positive feedback on their experiences of the priority-setting workshop. In particular, they valued the opportunity to express and share their views and potentially improve experiences for other young people. For example, young people commented, “[It] was an amazing opportunity to meet young people like myself and hear what they had to say while getting my own opinion voiced” and “It was a great experience and really fun to get together and talk about our experiences and how we could improve them for other young people”. Other stakeholders who attended the subsequent priority-setting workshops similarly provided positive feedback. In particular, stakeholders commented on the value of the opportunity to discuss youth public mental health with people from different backgrounds, job roles and sectors, and having young people involved as active participants alongside adults and hearing their views and experiences.

#### Peer support networks

Defining research priorities was the TRIUMPH Network’s first major task, and building relationships within the Network team, across sectors and disciplines, and between professionals and young people was an ongoing aspect of the priority-setting process. Building trusting relationships both between Network staff and YAG members, and between YAG members themselves, has been a priority, and significant staff time has been invested in these relationships. Discussing research priorities with YAG members has both benefited from these relationships and, in turn, strengthened the relationships as we learn more about each other and discover shared and differing experiences and views. While the Network had existing relationships with some stakeholders, the priority-setting process provided an opportunity to strengthen these relationships and develop new relationships with other individuals and organizations.

#### Blurring distinctions

A key strength is that the TRIUMPH Network prioritizes including views, ideas and experiences across stakeholder groups in all of our work. At the priority-setting workshops, we repeatedly heard that people have multiple intersecting professional and personal identities (for example, LGBTQ+ young people are also interested in other topics; some professionals are also under 24; some policy-makers also have lived experience of mental health issues). While these multiple identities were respected and explored in the TRIUMPH Network workshop discussions, as a Network, and in research more widely, these groups of stakeholders are often identified as being distinct. In the future, it is likely to be beneficial to approach transdisciplinary research that encompasses the intersectionality of people’s professional and personal identities, rather than requiring people to fit into specific stakeholder groups with certain expectations around their needs and experiences.

#### Facilitating rather than delivering

Romney [38] describes dialogue as “focused conversation, engaged in intentionally with the goal of increasing understanding”. In the process outlined in this paper, the conversation templates were designed to facilitate dialogue between the different stakeholders in order to hear each other’s thoughts, explore similarities/differences and arrive at a set of priorities that reflect the full range of experiences and viewpoints. However, despite its transdisciplinary nature, the TRIUMPH Network is located primarily within academia, and its focus is upon research as a means to improving youth mental health, whereas priorities for non-academic stakeholders were often related to development of practice. For example, where academics might want to ask questions about evidence, practitioners often wanted to ask questions about how a service should be provided and the funding available for it. Where this was the case, academic members of the team needed to make judgements about whether these practice-based priorities could be adapted to focus upon research. Therefore, while all stakeholders had the opportunity to engage in the focused conversation, they did not have the power to fundamentally change or meaningfully challenge the primary focus on research. While this dialogue shaped the final research priorities, the power to make final decisions about the priorities lay with the TRIUMPH Network theme leads (all academics) and the wider TRIUMPH Network team (mostly academics).

## Discussion

The research priorities identified in our study broadly support several of the areas previously identified by the McPin study [16]. For example, improving working relationships/links between organizations that support young people’s mental health, and training for school/college staff to better support young people’s mental health. However, given that the McPin study is focused towards treatment and support for mental health problems, and our study is focused towards promotion of good mental health and prevention of mental health problems, there are naturally differences in the findings. For example, the top two priority areas identified by the McPin study are concerned with the appropriateness of screening for early identification of mental health problems, and greater involvement of young people in decision-making about their mental health treatment [16]. As noted, the McPin study used the JLA PSP [17] to identify the Top 10 important areas for research. However, while the stages of the collaborative process are described [16], the reporting of the study does not provide reflections on, or an evaluation of, the process. Coproduction is a relatively new and innovative approach within mental health research. It is therefore important to put in place mechanisms to evaluate, measure or assess the impact of coproducing research in order to build up the evidence base around it [39]. Also, as mentioned, the McPin study was limited to priority areas and did not develop specific research questions. A separate stakeholder engagement exercise by NIHR, predominantly targeted at researchers, aligned the McPin priorities with specific research questions using the CHNRI method [19]. A sample of the Top 25 prioritized list of research questions have been validated by the McPin Young People’s Advisory Group. However, this list is currently confidential, and a detailed report of the methods and findings of the exercise has not yet been published, including any reflections on or evaluation or assessment of the process.

Similarly, several of the research themes identified by the Satellite study [22] are broadly consistent with priorities identified by the TRIUMPH Network. For example, the theme “lack of mental health literacy” is identified as a key factor that makes young people vulnerable to poor mental health, and the theme “enhance role of education system” is identified as a potential way to address this factor. However, the qualitative findings of the Satellite study are more problem- and solution-based compared to the findings of our study, possibly due to the difference in approach. Where the Satellite study takes a deficit-based approach, for example, identifying factors that increase children and young people’s vulnerability to poor mental health and potential ways to address them, our study takes a public mental health approach. Indeed, the Satellite study concludes that more research/evidence synthesis is required to identify interventions that move beyond an individual deficit-based approach to include broader factors such as peer and family support, and educational dimensions, which our study does. The Satellite study also does not provide reflections on, or an evaluation of, its approach to stakeholder engagement to inform a research agenda.

Our study also aligns to some extent with the four research challenges identified by the Emerging Minds Network. For example, the Supporting the Supporters challenge includes addressing how schools, as well as other settings, can support mental health promotion, prevention and treatment, and how young people themselves can help each other. However, the Emerging Minds Network is primarily focused on making a significant contribution to reducing the prevalence of mental health problems in children and young people, and this is particularly reflected in The Big Question challenge. The research priorities identified in our study are more focused on public mental health promotion and the thematic areas of Key Groups, Social Connections and Relationships, and Schools and Other Education Settings, and also focus on young people aged 10–24 years, whereas the Emerging Minds workshops involved a younger age range of children and adolescents.

By involving a range of stakeholders, including young people themselves, in defining the research priorities presented here, we hope that research based within these priority areas will have a positive impact on young people’s lives and improve their mental health. There is evidence that coproduction has positive outcomes [10], but few studies have explored how coproduction is achieved in research practice [12]. We therefore recommend that further work is undertaken to monitor and evaluate the impact of coproduction on both the research process and the research findings or outputs. There is an existing mental health study on the topic of technology to detect worsening mental health that reflects on the coproduction approach taken [40]. However, to the best of our knowledge, our study is the first to specifically provide reflections on a coproduction approach to defining research priorities for youth public mental health.

Researchers, practitioners and policy-makers all expressed that young people’s involvement in the priority-setting process was particularly powerful. For researchers, this collaboration from the beginning means that their research is informed by the needs of young people and is, therefore, more likely to be relevant and meaningful for young people. In addition, collaboration with practitioners and policy-makers means that research is informed by the context within which it exists and, therefore, has the best chance of being successful and making a positive impact. Having all these voices involved in the priority-setting process gave researchers a full range of stakeholder input and a wider view of the different perspectives in the research context.

One of the challenges in the process was finding a shared language related to youth public mental health research. Given the need for a full understanding of the context, it is vital that researchers hear practitioners’ and policy-makers’ frustrations at seeing research that seems not to have any impact on the situation in which they work, and that practitioners and policy-makers hear the limitations and possibilities for how research can support their work. While these conversations are not always easy, they resulted not only in a set of research priorities but also in ongoing collaborations based on shared interests and priorities.

The priority-setting process was the beginning of the TRIUMPH Network’s coproduction journey. Coproduction is not a one-off activity, and coproduction in the Network’s research priority-setting is intrinsically linked with ensuring that stakeholders inform all of the decisions related to the Network. Young people’s, and particularly the TRIUMPH YAG’s, involvement in setting the research priorities was the beginning of a conversation about what makes good research and the types of research areas that are of interest to young people that they would like to take forward. YAG members went on to assess funding proposals that were submitted to the Network based on the identified research priorities, and are currently undertaking their own research project that was informed by the research priorities. The thread of coproduction can clearly be seen through the setting of the research priorities, to young people’s involvement in setting the process for allocating funding, and young peoples’, practitioners’ and policy-makers’ involvement in assessing the funding applications. All funded projects were required to have research teams including non-academic partners and had to demonstrate how young people would be involved throughout the process.

### Challenges and limitations

There are challenges and limitations with this study. A key challenge, or tension, with the coproduction approach is that the three broad thematic areas, upon which the priority-setting process was based, were identified from the theme leads’ ongoing research and the literature, and were not coproduced with stakeholders. Further, other influences on young people’s mental health not covered within the three thematic areas did not form an intentional part of the workshop discussions. However, given the broad range of influences on young people’s mental health, these thematic areas provided a productive frame for stakeholder discussion. A limitation with the study is that while the United Kingdom-wide priority-setting workshops were open to all young people, those aged 10–12 years were not represented. In addition, workshop attendance was by invitation only to ensure a broad spread across disciplines; therefore, some relevant individuals or organizations may have been overlooked. A challenge with the United Kingdom-wide workshops was ensuring that young participants had the confidence to speak up. To address this challenge, YAG members gave a short presentation on constructive ways of working with young people at the start of the workshop, which we recommend in similar circumstances, such as “Keep it simple”, “Be kind” and “Don’t make assumptions: ask if young people understand”. The presentation included a pledge that all participants were asked to make: “We pledge to make sure that we do not talk over or ignore the views of young people today and that we respect all views, opinions, and experiences that are discussed. We will respect young people’s right to be listened to and for their views to inform decision-making that affects them.” Finally, although this paper has provided valuable reflections on the process from the perspective of the first four authors primarily involved, we recommend embedding a formal evaluation process in future work to support reflection from wider stakeholders involved, building on the coproduction approach, and as noted, to monitor and evaluate the impact of coproduction on the research process and research findings/outputs. We also recommend that a design-led approach, supportive of coproduction, is used to optimize the involvement of stakeholders and young people in particular, for example through the design of engaging tools such as the table-top conversation template.

The priorities identified in this work are of particular importance to inform the future work of the TRIUMPH Network, and are also relevant to the wider research, policy and practice communities working in the context of youth mental health. The priorities and reflections of the coproduction approach can be used to shape wider programmes and projects that seek to involve multiple stakeholders in research and to shape and inform the particular questions explored. We recommend that these learnings and reflections be considered and applied by others in future work.

## Conclusions

This paper contributes to the existing literature on health agenda-setting and collaborative research production, specifically research priorities in youth mental health. This paper describes a priority-setting process undertaken by the TRIUMPH Network to define research priorities for youth public mental health, and reflects on the coproduction approach taken to ensure the voices of multiple stakeholders, and importantly young people, were heard. The resultant set of research priorities and questions provides a focus for future research within youth public mental health and intervention development, and provides greater granularity than prior work on the thematic areas of Key Groups, Social Connections and Relationships, and Schools and Other Education Settings. Our transdisciplinary approach means that interventions targeting these priorities are more likely to be relevant to young people’s experiences and needs, and to fit with the needs of those working in practice and policy to support young people.

## Data Availability

The datasets are available from the corresponding author on reasonable request.

## Abbreviations

NIHR: National Institute for Health and Care Research
TRIUMPH: Transdisciplinary Research for the Improvement of Youth Mental Public Health
YAG: Youth Advisory Group
